# Intraportal Infusion of Ghrelin Could Inhibit Glucose-Stimulated GLP-1 Secretion by Enteric Neural Net in Wistar Rat

**DOI:** 10.1155/2014/923564

**Published:** 2014-08-26

**Authors:** Xiyao Zhang, Wensong Li, Ping Li, Manli Chang, Xu Huang, Qiang Li, Can Cui

**Affiliations:** ^1^Department of Endocrinology and Metabolism, The 4th Hospital Affiliated to Harbin Medical University, No. 37, Yiyuan Street, Harbin 150000, China; ^2^Department of Endocrinology and Metabolism, The 2nd Hospital Affiliated to Harbin Medical University, No. 246, Xuefu Road, Harbin 150080, China; ^3^Department of Laboratory Medicine, The 2nd Hospital Affiliated to Harbin Medical University, No. 246, Xuefu Road, Harbin 150080, China

## Abstract

As a regulator of food intake and energy metabolism, the role of ghrelin in glucose metabolism is still not fully understood. In this study, we determined the in vivo effect of ghrelin on incretin effect. We demonstrated that ghrelin inhibited the glucose-stimulated release of glucagon-like peptide-1 (GLP-1) when infused into the portal vein of Wistar rat. Hepatic vagotomy diminished the inhibitory effect of ghrelin on glucose-stimulated GLP-1 secretion. In addition, phentolamine, a nonselective α receptor antagonist, could recover the decrease of GLP-1 release induced by ghrelin infusion. Pralmorelin (an artificial growth hormone release peptide) infusion into the portal vein could also inhibit the glucose-stimulated release of GLP-1. And growth hormone secretagogue receptor antagonist, [D-lys3]-GHRP-6, infusion showed comparable increases of glucose stimulated GLP-1 release compared to ghrelin infusion into the portal vein. The data showed that intraportal infusion of ghrelin exerted an inhibitory effect on GLP-1 secretion through growth hormone secretagogue receptor 1α (GHS1α receptor), which indicated that the downregulation of ghrelin secretion after food intake was necessary for incretin effect. Furthermore, our results suggested that the enteric neural net involved hepatic vagal nerve and sympathetic nerve mediated inhibition effect of ghrelin on incretin effect.

## 1. Introduction

Ghrelin is a 28-amino acid peptide isolated from human and rat stomach as an endogenous natural ligand of GHS1α receptor (growth hormone secretagogue receptor 1α) [1]. Since first identified in 1999, ghrelin has been known as a multifaceted gut-brain peptide. Ghrelin stimulates food intake and fat deposition in adult animals [2] and humans [3]. Circulating ghrelin levels change under energy balance conditions, such as increasing with fasting, anorexia nervosa, or cachexia, and decreasing after food intake and in obesity [4–7]. Recent studies have shown that pretreatment with ghrelin exhibits protective and therapeutic effect in different organs, including stomach [8], gut [9, 10], pancreas [11], and even heart [12]. Ghrelin binds on GHS1α receptor to exert those effects. GHS1α is present in most of organs and tissues [13].

Although functions of ghrelin have been discovered more and more, as a gastric-intestine hormone, and secreted with a rhythm following food intake, the role of ghrelin on energy homeostasis is always a hot topic. Endogenous ghrelin has an important role in insulin secretion. Glucose-stimulated insulin secretion is reduced with exogenous ghrelin in humans [14] and rats [15]. On the other hand, it is reported that glucagon-like peptide-1 (GLP-1) reduces the rise in ghrelin levels in the late postprandial period at supraphysiological plasma levels through its insulinotropic action [16]. Interestingly, Radulescu et al. reported that different type of food intake resulted in different response of insulin, GLP-1, and ghrelin secretion [17]. The mechanism of those phenomena is still unclear. However, the changes of those hormones show that not one or two hormones but the gut-insular axis composed of a complicated hormone net regulates glucose metabolism to maintain the glucose levels in a narrow normal range.

In the study, we demonstrate the relationship of ghrelin and incretin effect. We investigate the change of glucose-stimulated GLP-1 concentration after exogenous ghrelin infused into the portal vein.

## 2. Materials and Methods

### 2.1. Animals

Normal male Wistar rats (age, 91 ± 8 d; body weight, 260 ± 21.3 g) were used in this study. All the rats were housed in wire-bottomed, stainless-steel cages and maintained in an ambient temperature of 20°C, with a light cycle between 0600 and 1800 h. The rats had free access to tap water and standard chow diet (Animal Nutrition Center, Harbin, China). The study was approved by the Ethics Committee of Harbin Medical University, China.

### 2.2. Surgery

All experiments were performed after anesthetizing the animals by intraperitoneally injecting 50 mg/kg pentobarbital sodium after a 12 h starvation period.

#### 2.2.1. Catheterization

After the induction of anesthesia was confirmed by the loss of the corneal reflex, polyethylene catheters (size, 0.5–1.0 mm) were inserted into the following veins: (1) left jugular vein for the collection of blood samples and (2) portal vein for the injection of agents.

#### 2.2.2. Hepatic Vagotomy

After the polyethylene catheter was placed into the portal vein, the hepatic branch of the anterior vagal trunk was either sectioned below the diaphragm or subjected to a sham operation as described in detail in the previous report [15].

### 2.3. Drug Treatments

#### 2.3.1. OGTT

After 30-minute rest after the surgery, glucose was administered through stomach catheter by 1 gram per kilogram (1 g/kg).

#### 2.3.2. Ghrelin Infusion

A dose of 1 ng/kg/mL of rat acylated (active) ghrelin (AG; Peptide Institute, Osaka, Japan) was pumped into the portal vein from 0 to 60 min at a rate of 1 mL/h by using a micropump after glucose administered by stomach catheter.

#### 2.3.3. KP-102 Infusion

KP-102 (GHRP-2, pralmorelin) (Peptide Institute, Osaka, Japan), a kind of artificial growth hormone release peptide, was infused at a dose of 1 *μ*g/kg/mL into the portal vein from 0 to 60 min with or without ghrelin (1 ng/kg/mL) after glucose administered by stomach catheter.

#### 2.3.4. Phentolamine Infusion

Phentolamine, a nonselective α receptor antagonist, was infused at a dose of 15 ng/kg/mL into the portal vein from 0 to 60 min with or without ghrelin (1 ng/kg/mL) after glucose administered by stomach catheter.

#### 2.3.5. GHS1α Receptor Antagonist Infusion

[D-lys3]-GHRP-6, GHS1α receptor antagonist, was infused at a dose of 30 ng/kg/mL into the portal vein from 0 to 60 min with or without ghrelin (1 ng/kg/mL) infused after glucose administered by stomach catheter.

### 2.4. Blood Sampling and Assays

Blood samples were drawn from the jugular vein at 0, 5, 10, 15, 30, and 60 min and immediately transferred into polypropylene tubes containing 1 mg/mL EDTA-2Na, aprotinin (final concentration, 500 kallikrein-inhibiting units (KIU)/mL), and DPP-4 inhibitor (10 *μ*g/mL). The plasma glucose level was measured using the glucose oxidase method (SINNOWA-D360PLUS, Jiangsu, China) after centrifuging the blood samples at 3000 rpm at 4°C for 10 min. The separated plasma was then stored at 4°C to assay the various parameters. The plasma insulin concentrations were measured by a radioimmunoassay (RIA) using the double-antibody technique, with rat insulin as the standard (Insulin RIA Kit; LINCO Research, St. Charles, Missouri, USA). The serum GLP-1 concentrations were measured using the RIA kits (active GLP-1 (7–36) RIA Kit; LINCO Research, St. Charles, Missouri, USA). At first, the samples were extracted by 95% alcohol. The sensitivity of this assay is 3 poml/L when a 100 *μ*L sample is used. The specificities of the assay to identify active GLP-1 (7–36) are 100% and the cross-reaction to GLP-1 (9–36) fragment was less than 1%.

### 2.5. Statistical Analysis

The parameters were compared among the groups with ANOVA and the Mann-Whitney *U* test using the SPSS 17.0 package (Chicago, IL, USA) for personal computers. A value of *P* < 0.05 (two-tailed) was regarded as significant. Different rats were used for each protocol (*n* = 8 for each protocol). The results represent the mean ± SE of the experiment values.

## 3. Results

### 3.1. GLP-1 Response to Ghrelin Administration in the OGTT

**Figure 1:**
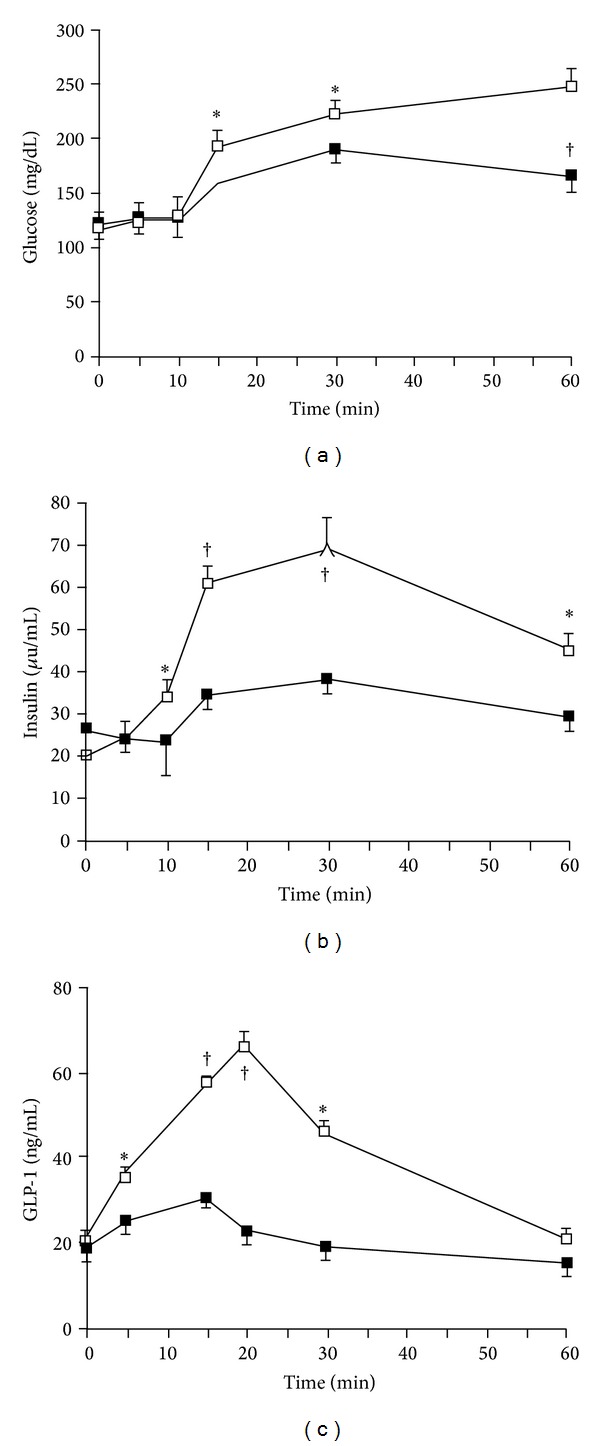
Plasma concentrations of glucose (a), insulin (b), and GLP-1 (c) during ghrelin infusion in the OGTT. Ghrelin (1 ng/kg/mL) was infused into the portal vein (i.p.) from 0 to 60 min at a rate of 1 mL/h using a micropump. A 20% glucose solution (1 g/kg) was administered through stomach catheter orally. Data are represented as mean ± SE. Open squares, saline i.p. group (*n* = 8); closed squares, ghrelin i.p. group (*n* = 8). *P < 0.05 versus saline i.p. group; ^†^ *P* < 0.01 versus saline i.p. group.

After glucose was loaded by stomach catheter, the plasma glucose concentrations were higher in the group of ghrelin infusion into the portal vein compared to those of saline infusion group (*P* values: <0.05, <0.05 and <0.01 at the time of 15, 30, and 60 min, Figure 1(a)). Correspondently, both the plasma insulin concentrations and plasma GLP-1 concentrations were lower when ghrelin was infused into the portal vein than when saline was infused (*P* values: <0.05, <0.01, <0.01, and <0.05 for insulin at the time of 10, 15, 30, and 60 min; *P* values: <0.05, <0.01, <0.01, and <0.05 for GLP-1 at the time of 5, 10, 15, and 30 min, resp.; Figures 1(b) and 1(c)).

### 3.2. The Role of the Visceral Vagus Nerve in GLP-1 Response to the Intraportal Infusion of Ghrelin in the OGTT

#### 3.2.1. The Effect of Hepatic Vagotomy on GLP-1 Response to the Intraportal Infusion of Ghrelin in the OGTT

**Figure 2:**
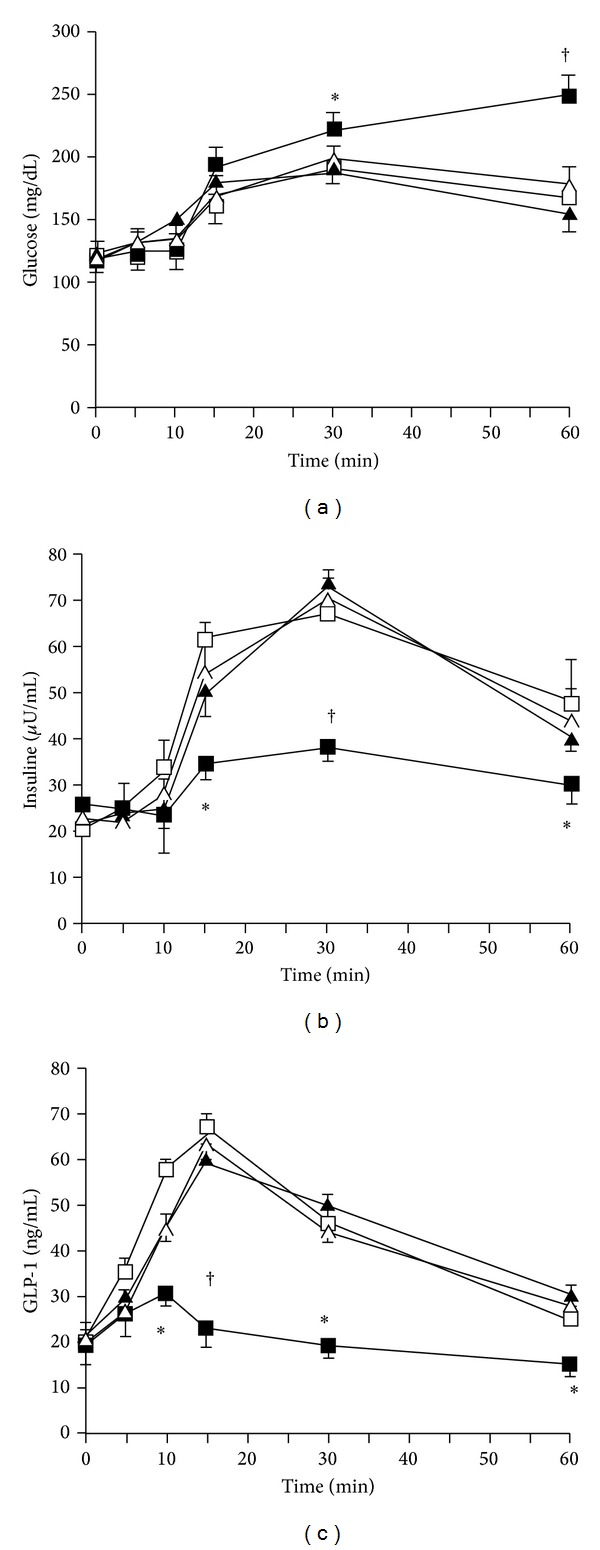
Plasma concentrations of glucose (a), insulin (b), and GLP-1 (c) during ghrelin infusion in the OGTT after hepatic vagotomy. After a 30 min rest after hepatic vagotomy or sham operation, ghrelin (1 ng/kg/mL) was infused into the portal vein (i.p.) from 0 to 60 min at a rate of 1 mL/h using a micropump. A 20% glucose solution (1 g/kg) was administered through stomach catheter orally. Data are represented as mean ± SE. Open squares, saline i.p. with sham operation group (*n* = 8); closed squares, ghrelin i.p. with sham operation group; open triangles, saline i.p. with hepatic vagotomy group (*n* = 8); and closed triangles, ghrelin i.p. with hepatic vagotomy group (*n* = 8). *P < 0.05 versus saline i.p. with sham operation group; ^†^ *P* < 0.01 versus saline i.p. with sham operation group.

When the hepatic branch of the anterior vagal trunk was sectioned, the insulin responses occurring at 10 and 15 min of OGTT were lower, and the plasma glucose levels were higher correspondingly (*P* < 0.05 for both; comparison between intraportal saline infusion after hepatic vagotomy and intraportal saline infusion in the sham operation group (Figures 2(a) and 2(b))). Plasma GLP-1 levels were also lower in the comparison between intraportal saline infusion after hepatic vagotomy and intraportal saline infusion in the sham operation group (*P* values: <0.05 and <0.05 at the time of 5 and 10 min; Figure 2(c)). In hepatic vagotomy group the inhibitory effect of ghrelin infusion into the portal vein on insulin and GLP-1 response in OGTT was diminished compared with the intraportal ghrelin infusion in the sham operation group (*P* values: <0.05; Figure 2(c)).

#### 3.2.2. The Effect of Phentolamine Infusion on GLP-1 Response to the Intraportal Infusion of Ghrelin in the OGTT

**Figure 3:**
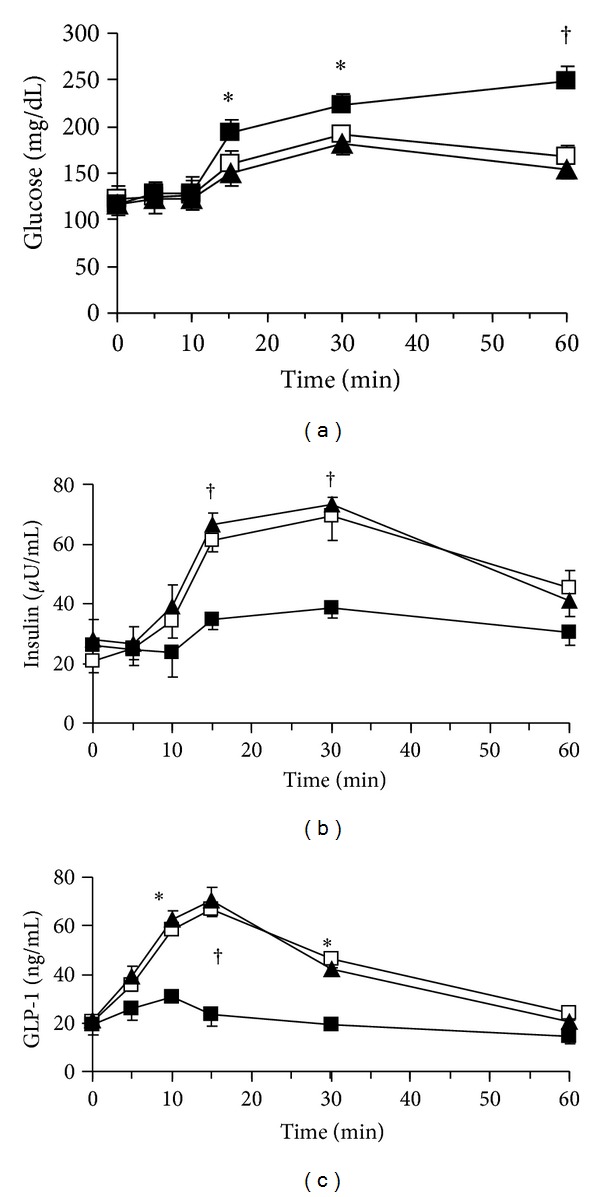
Plasma concentrations of glucose (a), insulin (b), and GLP-1 (c) during ghrelin infusion with or without phentolamine in the OGTT; phentolamine (15 ng/kg/mL) and ghrelin (1 ng/kg/mL) were infused into the portal vein (i.p.) from 0 to 60 min at a rate of 1 mL/h using a micropump. A 20% glucose solution (1 g/kg) was administered through stomach catheter orally. Data are represented as mean ± SE. Open squares, saline i.p. closed squares, ghrelin i.p.; and closed triangles, ghrelin with phentolamine i.p. *P < 0.05 versus, ghrelin i.p. group (*n* = 8); ^†^ *P* < 0.01 versus, ghrelin i.p. group (*n* = 8).

Phentolamine, a nonselective α receptor antagonist, infusion at 15 ng/kg/mL recovered the inhibitory effect of ghrelin on insulin and GLP-1 release in OGTT (*P* value <0.05, resp.; Figures 3(b) and 3(c)). To avoid the direct insulinotropic effect of phentolamine, we had screened for the appropriate dose of phentolamine at gradient of 15 mg/kg/m, 1.5 mg/kg/mL, 150 ng/kg/mL, 15 ng/kg/mL, and 5 ng/kg/mL. At last, we choose the dose of 15 ng/kg/mL, at which there is no direct effect on insulin secretion.

### 3.3. The Pathway of Ghrelin on GLP-1 Response in the OGTT

#### 3.3.1. GLP-1 Response to KP-102 Administration in the OGTT

**Figure 4:**
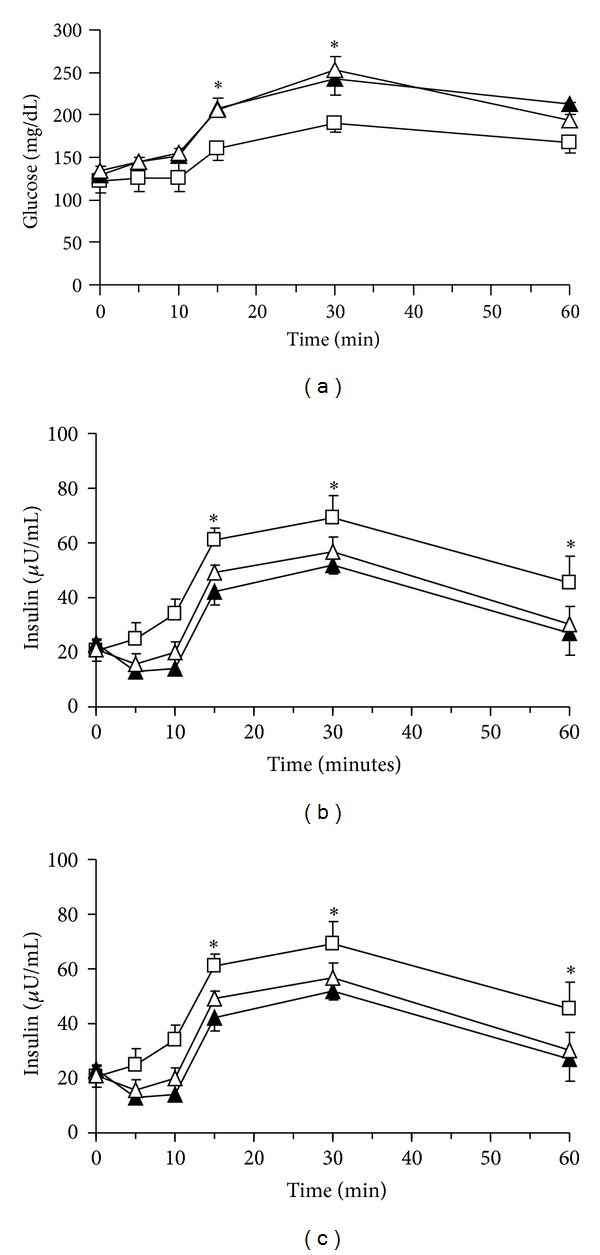
Plasma concentrations of glucose (a), insulin (b), and GLP-1 (c) during KP-102 infusion with or without ghrelin in the OGTT. KP-102 (1 *μ*g/kg/mL) and ghrelin (1 ng/kg/mL) were infused into the portal vein (i.p.) from 0 to 60 min at a rate of 1 mL/h using a micropump. A 20% glucose solution (1 g/kg) was administered through stomach catheter orally. Data are represented as mean ± SE. Open squares, saline i.p. group (*n* = 8); closed triangles, KP-102 i.p. group (*n* = 8); open triangles, KP-102 with ghrelin i.p. group (*n* = 8). *P < 0.05 versus saline i.p.; ^†^ *P* < 0.01 versus saline i.p. with sham operation group.

After glucose was loaded by stomach catheter, there were great changes of the plasma glucose concentrations by the KP-102 infusion into the portal vein at the dose of 1 *μ*g/kg/mL (Figure 4(a)). The plasma insulin concentrations and plasma GLP-1 concentrations were lower, consistent with the time point plasma glucose increases (*P* values: <0.05, <0.01, <0.01, <0.05, and <0.05 for insulin; *P* values: <0.05, <0.01, <0.01, 0.01, and <0.01 for GLP-1 at 5, 10, 15, 30, and 60 min, resp.; Figures 4(b) and 4(c)). Neither plasma insulin concentrations nor GLP-1 concentrations were far lower when KP-102 plus ghrelin infused into portal vein than when KP-102 alone was infused (*P* values: >0.05; Figures 4(b) and 4(c)).

#### 3.3.2. The Effect of GHS1α Receptor Antagonist Infusion on GLP-1 Response to the Intraportal Infusion of Ghrelin in the OGTT

**Figure 5:**
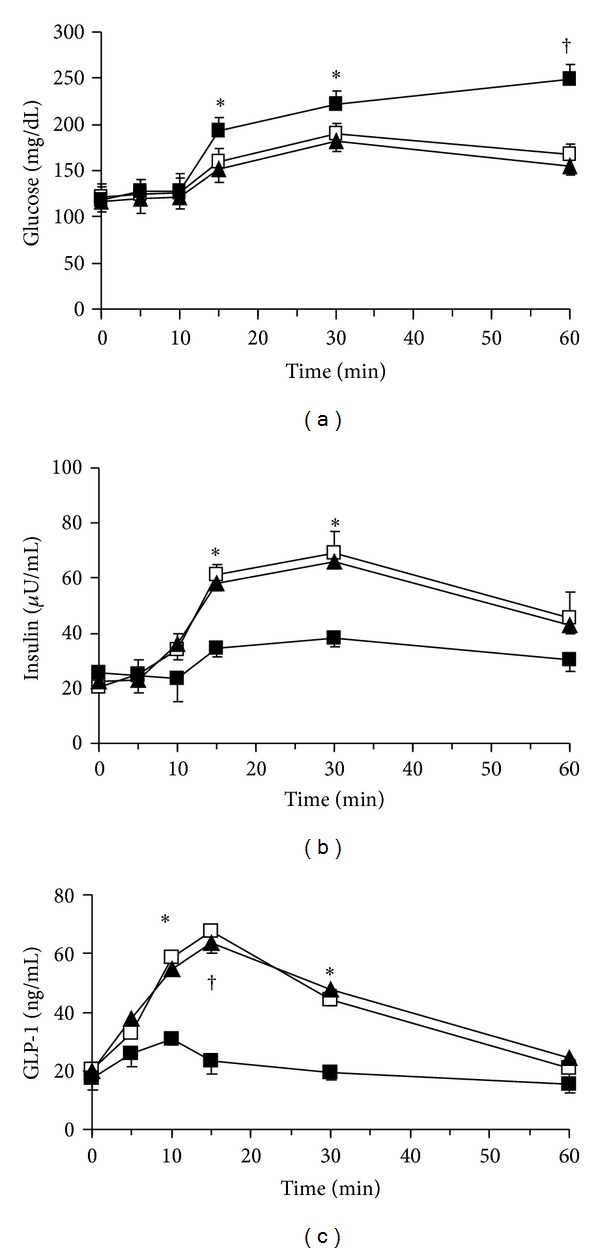
Plasma concentrations of glucose (a), insulin (b), and GLP-1 (c) during ghrelin infusion with or without GHRP-6 in the OGTT; GHRP-6 (30 ng/kg/mL) and ghrelin (1 ng/kg/mL) were infused into the portal vein (i.p.) from 0 to 60 min at a rate of 1 mL/h using a micropump. A 20% glucose solution (1 g/kg) was administered through stomach catheter orally. Data are represented as mean ± SE. Open squares, saline i.p. closed squares, ghrelin i.p.; and closed triangles, ghrelin with GHRP-6 i.p. *P < 0.05 versus, ghrelin i.p. group (*n* = 8); ^†^ *P* < 0.01 versus, ghrelin i.p. group (*n* = 8).

Ghrelin coinfusion with [D-lys3]-GHRP-6, GHS1α receptor antagonist, at the dose of 30 ng/kg/mL did not induce the inhibitory effect on insulin and GLP-1 response compared with the intraportal ghrelin infusion alone group (*P* values: <0.05, resp.; Figures 5(b) and 5(c)).

## 4. Discussion

In the previous study, the acute infusion of ghrelin from portal vein actively inhibited the glucose-stimulated insulin secretion in IPGTT in rats [15]. The results of present study confirmed the exogenous ghrelin inhibited glucose-stimulated insulin secretion in OGTT in rats. With insulin decrease, the decrease of plasma GLP-1 after oral glucose load was also seen. The results showed that ghrelin infused into the portal vein could affect incretin effect. It is known that plasma ghrelin and insulin levels are inversely related during the fasting and postprandial states [4]. Low ghrelin levels are independently associated with insulin resistance and type 2 diabetes [18]. Therefore, we deduced that high level of ghrelin on fasting and its rapid descending after food intake was indispensable for the occurrence of the incretin effect. If such secretion rhythm loses, the incretin effect might be impaired, just as what happens in type 2 diabetes.

The hepatic portal system is closely related to insulin secretion and glucose metabolism, including incretin effect. It is known that the vagus nerve organizes the hepatic portal system and the afferent firing of the vagus nerve is mediated by the glucose concentration in the portal vein. We have observed that exogenous ghrelin inhibited the glucose-induced insulin secretion via its action on the vagus nerve in IPGTT [5]. To explore the possible role of vagus nerve in the downregulation of GLP-1 secretion observed in the present study, possible contributions of the enteric nervous system were abolished by transection of hepatic vagus nerve. After hepatic vagotomy, the inhibitory effect of ghrelin on GLP-1 secretion was diminished, which suggested that hepatic vagus nerve was closely related to incretin effect. After meal or oral glucose test, activation of vagal afferents may induce the secretion of GLP-1 and disruption of afferent vagal fibers by hepatic vagotomy resulted in a loss of the rapid L cell response to ingested nutrients [19]. The ghrelin signal released in the portal vein might block the role of oral glucose on the hepatic branch of afferent vagus nerve and diminish its ability to induce incretin effect. Thus, the downregulation of ghrelin might be required for the incretin effect after a meal.

The functioning of the neuroendocrine loop mediates proximal nutrients induced GLP-1 secretion [19]. In present scenario, phentolamine coinfusion with ghrelin into the portal vein also recovered the inhibitory effect of ghrelin on GLP-1 secretion. Phentolamine, as an α receptor blocker, is also a strong insulinotropic. To avoid the direct insulinotropic effect of phentolamine, we had screened for the appropriate dose of phentolamine at gradient as described in Methods.

Ghrelin regulates GLP-1 secretion in portal system through its GHS1α receptor. The evidences were provided by the data of KP-102 and [D-lys3]-GHRP-6 administered groups. KP-102 infused into the portal also inhibited glucose stimulated GLP-1 secretion, which indirectly approved that ghrelin mediated GLP-1 secretion through its receptor GHS1α on hepatic vagus nerve. As a kind of artificial growth hormone release peptide, GHS1α was the only known receptor of KP102. Furthermore, the result that [D-lys3]-GHRP-6, GHS1α receptor antagonist, coinfusion with ghrelin recovered the inhibitory effect of ghrelin on GLP-1 secretion gave a direct evidence.

Many studies using animal models selected the time of OGTT longer (for example, until 120–150–180 minutes) than 60 minutes. We explained the choice of time to 60 minutes for OGTT and ghrelin infusion. First, in the study, we tested GLP-1 levels, which need relatively more blood samples for assay (1 mL for each point). If we take more time points to 120 minutes, even 180 minutes, the blood sample would not have been enough for a rat. Some rats could not last to 120 min or longer. Second, the animal selected in the study was in normal glucose tolerance and our results showed that 60 min of OGTT was enough to explain the changes of glucose, insulin, and GLP-1 by exogenous ghrelin infusion, especially for GLP-1. The time point selected is enough to reflect the physical changes.

In the prestudy, we had selected the glucose dose for OGTT. We found that, administering glucose at the dose of 1 g/kg compared to dose of 2 g/kg, there is no obvious difference for the results of plasma glucose and insulin levels. The mean body weight of rats in our study is about 250 g, not fat, so we choose the low dose. To avoid the direct insulinotropic effect of phentolamine, we had screened for the appropriate dose of phentolamine at gradient of 15 mg/kg/m, 1.5 mg/kg/mL, 150 ng/kg/mL, 15 ng/kg/mL, and 5 ng/kg/mL. At last, we choose the dose of 15 ng/kg/mL, at which there is no direct effect on insulin secretion. Phentolamine is a nonselective α receptor antagonist. Before the test, we do not know what result we could get. We think that it is better to choose a nonselective antagonist. In the later study, we plan to choose a selective one.

In summary, we observed that exogenous ghrelin inhibited incretin effect through the portal enteric nerve loop. The high concentration of ghrelin on fast and rapid downregulation after meal is one of a switch to initiate incretin effect. The functions of neuroendocrine loop in gut-insulin axis may be very helpful for understanding the role of this axis in pathogenesis of type 2 diabetes.
